# Large language model use in clinical oncology

**DOI:** 10.1038/s41698-024-00733-4

**Published:** 2024-10-23

**Authors:** Nicolas Carl, Franziska Schramm, Sarah Haggenmüller, Jakob Nikolas Kather, Martin J. Hetz, Christoph Wies, Maurice Stephan Michel, Frederik Wessels, Titus J. Brinker

**Affiliations:** 1 Department of Digital Prevention, Diagnostics and Therapy Guidance, German Cancer Research Center (DKFZ), Heidelberg, Germany; 2 Department of Urology and Urological Surgery, University Medical Center Mannheim, Ruprecht-Karls University Heidelberg, Mannheim, Germany; 3 Else Kroener Fresenius Center for Digital Health, Medical Faculty Carl Gustav Carus, Technical University Dresden, Dresden, Germany; 4 Medical Faculty, Ruprecht-Karls University Heidelberg, Heidelberg, Germany

**Keywords:** Cancer, Mathematics and computing

## Abstract

Large language models (LLMs) are undergoing intensive research for various healthcare domains. This systematic review and meta-analysis assesses current applications, methodologies, and the performance of LLMs in clinical oncology. A mixed-methods approach was used to extract, summarize, and compare methodological approaches and outcomes. This review includes 34 studies. LLMs are primarily evaluated on their ability to answer oncologic questions across various domains. The meta-analysis highlights a significant performance variance, influenced by diverse methodologies and evaluation criteria. Furthermore, differences in inherent model capabilities, prompting strategies, and oncological subdomains contribute to heterogeneity. The lack of use of standardized and LLM-specific reporting protocols leads to methodological disparities, which must be addressed to ensure comparability in LLM research and ultimately leverage the reliable integration of LLM technologies into clinical practice.

## Introduction

In 2022, the artificial intelligence (AI) scene was revolutionized with the introduction of ChatGPT^1^, a freely accessible large language model (LLM) trained to generate human-like text and perform complex natural language processing tasks. Research on AI in medicine has rapidly expanded, as evidenced by a doubling in publication volume from 2015 to 2022, a surge in venture capital investment from USD 2.7 billion to USD 12.2 billion between 2018 and 2021, and rich methodological diversity in applying AI to enhance diagnostics and patient care^2^. Following the success of ChatGPT developed by OpenAI^©^, many other companies have launched their own LLMs, such as Microsoft^©^ (e.g. Copilot^3^), Google (e.g. Gemini^4^), and Meta^©^ (e.g. LLaMA^5^). LLMs, primarily ChatGPT, have been investigated for their potential to improve various aspects of oncology, such as patient information provision, therapy management, and response prediction from clinical notes. These models can handle tasks like content generation, language translation, and medical question-answering^6^. This has the potential to provide accessible information to patients and to facilitate seamless communication between clinicians, thereby enhancing patient empowerment and satisfaction^7^. Despite their capabilities, LLMs in oncology have demonstrated significant error rates and a tendency to provide obsolete data^6,7^. Advanced AI tools show both promising opportunities and limitations in the use of LLMs in oncology^6^. The aforementioned most recent reviews provide a comprehensive overview as of March and September 2023, yet without a meta-analysis of LLM performance^6,7^. Since then, many new studies have further investigated the use of large language models in oncology, covering various application domains, types of cancer, medical tasks, and have presented a broad range of methodological approaches.

From a computational perspective, LLMs are predominantly based on transformer architectures. As outlined by Perez-Lopez et al., transformers have achieved promising results in the realm of multimodal AI—models designed to integrate and analyse multiple types of data, such as text and images—by effectively handling complex medical datasets^8^. Additionally, Truhn et al. illustrated that both text and image processing networks are increasingly adopting transformer neural networks^9^. In conclusion, the development of multimodal AI, driven by transformer architectures, marks a significant qualitative shift from the specialized niche models that were prevalent in the 2010s^9^.

Given the mixed outcomes observed with the implementation of LLMs in oncology and the rapid evolution in this field of research, this systematic review aims to:

1. Conduct a comprehensive analysis and overview of the current literature on the applications of LLMs in oncology.
2. Perform a formal meta-analysis to quantitatively assess and illustrate the reported correctness of studies evaluating LLMs in medical question-answering.
3. Analyse the methodologies employed and outline constraints that should be addressed in future research.

## Methods

### Protocol and registration

The PRISMA^10^ and QUADAS-2^11^ guidelines were used as orientation since no specific AI-related reporting guidelines for systematic reviews exist currently. The review protocol was registered in PROSPERO prior to study commencement (registration number: CRD42024529996)^12^.

### Search strategy

Two independent reviewers (N.C., F.S.) searched the PubMed database (including MEDLINE) using the following search query (last accessed 19 March 2024): “((LLM) OR (Large Language Model) OR (ChatGPT)) AND ((oncology) OR (cancer))”. The term “ChatGPT” was incorporated due to it being the most prominent example of LLMs presently. This inclusion aimed to prevent the exclusion of relevant literature that pertained to LLMs but might have referred specifically to “ChatGPT” rather than using the term “LLM” itself. For a detailed overview of the comprehensive search strategy (see the PRISMA checklist in Supplementary Table 1).

### Study selection

Selection criteria were defined a priori to identify relevant studies for inclusion in this systematic review. Eligible studies were required to have an available abstract, focus on the application of LLMs in oncology, and be original research articles published in peer-reviewed journals in English between 2021 and 2024. The exclusion criteria for this scientific review included studies that did not address LLM in oncology, lacked rigorous methodology, preprints, systematic reviews, non-peer-reviewed articles or opinion pieces, published in languages other than English, or did not have available full texts.

Two independent reviewers (N.C., F.S.) screened the titles and abstracts of the retrieved records to identify potentially relevant studies. The full-text articles of these studies were then assessed for inclusion using the predefined criteria. Any discrepancies between the reviewers regarding study eligibility were resolved through discussion or consultation with a third reviewer (S.H.).

### Study analysis

Data analysis of the included studies was conducted independently by the same reviewers (N.C., F.S.) using a standardized form. The included studies were analysed for the following information: general study characteristics (authors, year of publication), name of the LLM or alternative tool used, comparison group, study population, intervention details, and outcomes measured. The collected information was organized and synthesized according to the following characteristics: LLM, cancer entity, medical task, application domain (as per the classification proposed by Clusmann et al.^7^), comparison type, and evaluation metrics employed in Supplementary Table 2. An evaluation framework consisting of 18 Items was created (Table 1).

**Table 1:** Evaluation framework used for the systematic data extraction of studies evaluating the performance of LLM in medQA

Section	Item	Description
Source of prompts	1	Website, exam question bank, FAQs (Google Trends **©**), guidelines, patient information forum
	2	Number of questions (*n*)
Assessed large language model	3	Which LMM (*e.g. GPT-3.5, Gemini*)?
	4	Standard model or fine-tuning with specific data applied?
Questioning procedure	5	Topic (*e.g. cancer entity*)
	6	Source data of prompts and answers provided?
	7	Prompt-engineering used?
	8	Enquiry conducted once or repeated?
	9	Enquiry conducted independent (*i.e. “new question* *=* *new chat”*)
	10	Standalone questions or multiple continuous questions (*i.e. “zero-shot” vs. “fire-side” enquiry*)
	11	Language
Output evaluation	12	Rater (*Who is evaluating LLM output and level of experience?*)
	13	Is the rater blinded?
	14	Number of raters?
	15	If multiple raters, is inter-rater agreement reported?
	16	Is there an endpoint as reported as metrics? *(e.g. accuracy, readability)*
	17	Is grading of LMM output reported? *(e.g. binary yes/no, Likert-scale, multidimensional?)*
	18	Is a control group reported? (*e.g. “We compared performance of GPT-3.5 with GPT-4”*)

### Statistical analysis and meta-analysis

Publications investigating the application domain “medical knowledge” using LLMs in a prompt-answering principle were included in the meta-analysis. Publications were eligible if the total number of questions and an endpoint that describes the relative amount of correct answers in percentages were reported (usually reported as *accuracy* or *sensitivity*). Using a random-effects model, the *I*² statistic was calculated using the R-package *metafor*^13^. Due to high heterogeneity, the risk of bias or quality of evidence evaluation was not estimated. All statistical analyses were performed using RStudio (Version 2024.04.0+735). However, it is worth noting that no formal hypothesis testing was conducted. Therefore, the presented meta-analysis is of a descriptive nature.

## Results

### Systematic review

In the conducted literature search, initially, 483 publications matched the search string. During the title and abstract screening, 373 publications were excluded. 110 publications were full-text screened, resulting in the exclusion of another 76 publications. Finally, this systematic review includes 34 eligible studies, all of which were published between January 2021 and March 2024. For a detailed overview of the study, the selection is presented as a PRISMA flow diagram (see Supplementary Fig. 1). For a comprehensive overview of the included publications in this systematic review (see Tables 2 and 3).

**Table 2:** Examples of prompt engineering strategies reported by authors

Publication	LLM	Question type	Prompt design	Outcome
Schulte (2023)^15^	GPT-3.5	OE	First describing clinical scenarios and afterwards prompting to create a list of possible therapies in respect to metastatic solid tumours. Template: *“[X] … name combined or single agents for systemic therapies that would be administered as first line treatment?”*	77% of named therapies are in concordance with guidelines across all evaluated tumour entities.
Chen et al. (2023)^17^	GPT-3.5	OE	Various question styles were evaluated by altering prompt syntax. All 4 versions were evaluated per cancer entity. Templates: *1.“[What is the treatment for [X]?”* *2. “[What is a recommended treatment for [X] according to NCCN?”* *3. “[What is a recommended treatment [X]?”* *4. “[How do you treat [X]?”*	Prompt style had an influence on the number of treatments recommended in accordance with the NCCN guidelines, but also on the number of hallucinated, i.e., false treatment recommendations.
Sorin et al. (2023)^20^	GPT-3.5	OE	Asking LLM to recommend the next most appropriate step in management and providing detailed patient history as a basis for decision Template: *“Hi, can I give you a patient story of breast cancer detected, and you’ll say what is the next step in her management? Please decide if she needs surgery, what type of surgery, whether she needs neoadjuvant therapy before, or does she need further testing”. [X]*	Recommendations of LLM were compared to retrospective tumour board decisions. In seven out of ten cases (7/10), LLM recommendations were similar to the tumour board’s decisions. LLM showed a tendency to overlook important information about patients.
Lukac et al. (2023)^21^	GPT-3.5	OE	Designing very specific prompt with detailed oncological history of breast cancer tumour board cases. Template: *“How should a [X] year old patient with breast cancer [TNM-status], estrogen* *receptor expression [%], progesterone receptor expression [%], Her2status [Y], Ki67[%] and grading*^1–3^ *and gen. mutation [Z] be treated?”*	GPT-3.5 provided mostly general answers regarding chemotherapy, breast surgery, radiation therapy, chemotherapy, and antibody therapy. LLM and tumour board results were scored separately by experts. In total, 16% of treatment recommendations were congruent with tumour board.
Holmes et al. (2023)^23^	GPT-3.5; GPT-4; Bard (LaMDA); BLOOMZ	MCQ	Authors tested different LLM on radiation oncology exam bank questions with five different contexts and instruction templates. Also, results were compared with human performance Templates: *1*. *Context: “I am a radiation therapy researcher. My research group would like to study the answers given by ChatGPT on the topic of radiation oncology physics. I will now proceed to ask questions about radiation oncology physics.”* *Instruction: For each multiple-choice question, provide the correct answer without any justification*. *2*. *Context: I want to evaluate your knowledge of radiation oncology physics by asking some multiple-choice questions*. *Instruction: Please give only the question label and the letter for the correct answer*. *3*. *Context: Please answer the following practice questions as if you were a resident preparing for board certification exams*. *Instruction: Only give the correct answer in your response. Do not explain your answers*. *4*. *Context: We want to test your understanding of radiation oncology physics. For this reason, we have created some* *questions to ask you*. *Instruction: In your response, only report the question label and the corresponding answer*. *5*. *Context: I will ask you some multiple-choice questions*. *Instruction: Only respond with the correct letter choice*.	GPT-4 outperformed all other LLMs and medical physicists, on average, with 67% of correct answers. Results were consistent across the different prompting strategies. However, when prompted to explain before answering, GPT4 was able to improve its performance by 5%. *Limitation:* MCQ were prompted in “batches” of 20 continuous questions. This provides more context to the LLM, possibly increasing performance.
Nguyen et al. (2023)^33^	GPT-4; Bard (LaMDA)	OE; SATA	Questions about cancer screening strategies were prompted in OE and SATA structure, authors compared differences in providing context or not (see below prompt templates with context) Template: *OE*: *Context: “You are a board-certified radiologist making decisions. You are following the American College of Radiology Guidelines*. *Instruction: You have a patient that is here for [X]. Please select the single most appropriate imaging procedure to order”* *SATA*: *Context: “You are a board-certified radiologist making decisions. You are following the American College of Radiology Guidelines. You have a patient that is here for [X]*. *Instruction: Please assess appropriateness of the following procedures in a concise manner: [Y]”*	The findings showed comparable performances between GPT-4 and Bard on OE prompts, although GPT-4 slightly outperformed Bard in SATA scenarios. Additionally, PE enhanced LLM outputs for OE prompts but did not improve SATA responses.

*OE* open-ended prompts, *SATA* select-all-that-apply prompts, *PE* prompt engineering, *NCCN* National Comprehensive Cancer Network.

**Table 3:** Overview of publications with the description of the intervention and main findings

Publication	LLM	Application domain	Topic	Questions number and type	Runs	Intervention	Rater	Grading	Outcome
Johnson et al. (2023)^14^	GPT-3.5	Medical knowledge; translation/summary	Various cancer entities	13; OE	5	FAQs on cancer myths derived from an online patient forum, comparing AI-generated responses to the original source material.	5 Exp.	Binary accuracy (yes/no); Readability (FKGL*)	LLM accuracy: 0.969; NCI accuracy: 1.0. LLMs consistently answered repetitive questions accurately. Both sources had lower readability levels than health literacy guidelines recommend.
Schulte (2023)^15^	GPT-3.5	Medical knowledge	Metastatic solid tumours	51; SATA; PE	1	Prompted the LLM to list therapies and compared the total recommended treatments to NCCN guidelines.	Author	“Valid Therapy Quotient” (VQT)	VQT of 0.77; 77% of named therapies aligned with guidelines.
Coskun et al. (2023)^16^	GPT-3.5	Medical knowledge	Prostate cancer	59; OE	NR	FAQs on prevention, aetiology, diagnosis, prognosis, and therapy from the official European Association of Urology (EAU) patient forum, compared to the reference source.	2 Exp.	Multidimensional: Accuracy includes true positive (TP), false positive (FP), true negative (TN), and false negative (FN). Similarity is measured by cosine similarity.	Precision: 0.426; Recall: 0.549; F1 Score: 0.426; Cosine similarity: 0.609; Mean GQS: 3.62.
Chen et al. (2023)^17^	GPT-3.5	Medical knowledge	Lung; prostate; breast cancer	104; OE; PE	NR	Evaluated treatment prompts in four styles against NCCN guidelines.	3 Exp.	Multidimensional: Uses a self-developed 5-item grading system to assess the number of recommended treatments and their concordance with guidelines.	LLM provided at least one NCCN-concordant treatment: 1.0; non-concordant treatment: 0.343; hallucinated responses: 0.125; interrater agreement: 0.62.
Lombardo et al. (2024)^18^	GPT-3.5	Medical knowledge	Prostate cancer	195; OE	NR	Prompts were drafted from GL recommendations on Classification; Diagnosis; Treatment; Follow-Up; QoL and compared to reference source; assessed on correctness	2 Exp.	4-point Likert-scale: 1—Completely correct; 2—Correct but inadequate; 3—Mix of correct and misleading; 4—Completely incorrect.	Completely correct: 0.26; Correct but inadequate: 0.26; Mix of correct and misleading: 0.24; Incorrect: 0.24; Best performance in follow-up and QoL; Worst performance in diagnosis and treatment
Ozgor et al. (2024)^19^	GPT-4	Medical knowledge	Genitourinary cancer	210; OE	NR	FAQs on diagnosis; treatment; aetiology; follow-up from various sources vs. prompts derived from GL	3 Exp.	5-point Likert-scale Quality; Global Quality Score (GQS); 5 = highest	GQS score of 5 for prostate cancer: 0.646; for bladder cancer: 0.629; for kidney cancer: 0.681; and for testicular cancer: 0.639; Mean GQS score of towards GL questions was significantly lower than answers given to FAQs. Performance to questions aligned with the EAU guideline was deemed unsatisfactory.
Sorin et al. (2023)^20^	GPT-3.5	Medical knowledge	Breast cancer	10; OE	NR	LMM was given a detailed patient history and prompted to recommend treatment. Recommendations of LLM were compared to retrospective tumour board decisions.	2 Exp.	Multidimensional: 5-point Likert-scale for agreement with TB; summarization and explanation.	Agreement with TB 70%; Mean scores for the first reviewer were summarization: 3.7; recommendation: 4.3; and explanation: 4.6; Mean scores for the second reviewer were summarization: 4.3; recommendation: 4.0; and explanation: 4.3; LLM showed tendency in some cases to overlook important information about the patient
Lukac et al. (2023)^21^	GPT-3.5	Medical knowledge	Breast cancer	10; OE	NR	LMM was given a detailed patient history and prompted to recommend treatment. Recommendations of LLM were compared to retrospective tumour board decisions.	NR	Agreement (point-based scale 0–2)	0.16 of outputs congruent with TB. LLM provided mostly generalized answers; the current version is not able to provide specific recommendations for the therapy of patients with primary breast cancer.
Gebrael et al. (2023)^22^	GPT-4	Medical knowledge	Metastatic prostate cancer	56; OE	NR	LMM was given a detailed patient history of patients presented to the emergency ward with metastatic prostate cancer; LLM was prompted to decide to discharge or admit.	NR	Sensitivity and specificity of GPT-4 in determining whether a patient required admission or discharge.	LLM sensitivity in determining admission: 0.957; LLM specificity in discharging patients: 0.182. Findings suggest that GPT-4 has the potential to assist health providers in improving patient triage in emergency
Holmes et al. (2023)^23^	GPT-3.5; GPT-4; Bard; BLOOMZ	Medical knowledge	Radiation oncology	100; MCQ	5	Authors tested different LLM on radiation oncology exam bank questions with 5 different Context and Instruction templates. Also; results were compared with human performance.	9 Exp.; 6 non-Exp.	Accuracy (number of correct responses)	GPT-4 outperformed all other LLMs and medical physicists, on average; with 67% of correct answers.
Haver et al. (2023)^46^	GPT-3.5; GPT-4; Bard	Translation/summary	Lung cancer	19; OE	3	Evaluated the use of three LLMs for simplifying LLM-generated responses to common questions about lung cancer and lung cancer screening.	3 Exp.	Readability FRE*; FKRG*	GPT-3.5’s baseline responses to lung cancer and LCS questions were challenging to read. Simplified responses from all three LLMs (GPT-3.5, GPT-4, Bard) enhanced readability, with Bard showing the most improvement. However, the average readability of these simplified responses still exceeded an eighth-grade level, too complex for the average adult patient.
Choo et al. (2024)^24^	GPT-3.5	Medical knowledge	Colorectal cancer	30; OE	NR	LMM was given a detailed patient history and prompted to recommend treatment. Recommendations of LLM were compared to retrospective tumour board decisions.	NR	4-point Likert-scale; Concordance with TB	Results deemed satisfactory; with concordance between LLM and tumour board 0.733. LLM recommendations did not match TB in 0.13
Haemmerli et al. (2023)^25^	GPT-3.5	Medical knowledge	Brain cancer	10; OE	NR	LLM was prompted with a detailed patient history to recommend treatment, which was then evaluated by a rater. Interrater agreement was also assessed.	7 Exp.	10-point Likert scale used to rate agreement with LLM recommendations; intraclass correlation coefficient (ICC) measured interrater agreement.	LMM median responses: diagnosis—3, treatment—7, therapy regimen—6, overall agreement—5. Performance was poor for classifying glioma types, but good for recommending adjuvant treatments. Overall, there was moderate expert agreement, with an ICC of 0.7.
Griewing et al. (2023)^26^	GPT-3.5	Medical knowledge	Breast cancer	20; OE	NR	LLM was provided a detailed patient history and prompted to recommend treatments, which were then compared to retrospective tumour board decisions. The cases were designed to showcase the pathological and immune morphological diversity of primary breast cancer.	13 Exp.	Number of treatment recommendations and concordance with TB	LLM proposed 61 treatment recommendations compared to 48 by experts, with the largest discrepancy in genetic testing. Overall concordance between LLM and experts was 0.5. LLM was deemed inadequate as a support tool for tumour boards.
Benary et al. (2023)^27^	GPT-3.5; perplexity; BioMedLM; Galactica	Medical knowledge	Various cancer entities	10; OE	NR	Cases of advanced cancer with genetic alterations were submitted to four LLMs and one expert physician for personalized treatment identification. The concordance of LLM-generated treatments with the human reference was evaluated.	1 Exp.	Categories: true positive (TP), false positive (FP), true negative (TN), false negative (FN). Likelihood of a treatment option originating from an LLM rated on a Likert-scale from 0 to 10	LLMs proposed a median of 4 treatment recommendations with F1 scores of 0.04, 0.17, 0.14, and 0.19 across all patients. LLMs failed to match the quality and credibility of human experts.
Davis et al. (2023)^28^	GPT-3.5	Medical knowledge; summary/translation	Oropharyngeal	15; OE	NR	LLM outputs assessed for accuracy; comprehensiveness; and similarity. Readability assessed. Authors developed a new Score. Responses graded lower than an average of 3 were commented by raters.	4 Exp.	Multidimensional: 5-point Likert-scales for Accuracy;Comprehensiveness; Similarity; Readability (FRE*; FKGL*)	LLM responses were suboptimal, with average accuracy: 3.88; comprehensiveness: 3.80; and similarity: 3.67. FRE and FKRGL scores both indicated higher than the 6th-grade level recommended for patients. Physician Comments: suboptimal education value and potential to misinform.
Atarere et al. (2024)^29^	GPT-3.5; YouChat; Copilot	Medical knowledge	Colorectal	20; OE	3	5 questions on important colorectal cancer screening concepts and 5 common questions asked by patients about diagnosis and treatment. LLM outputs compared to GL as a reference	2 Exp.	Binary for 2 dimensions: appropriateness (yes/no); reliability (yes/no)	GPT-3.5 and YouChat reliably appropriate responses for screening: 1.0; Copilot reliably appropriate responses for screening: 0.867; GPT-3.5 reliably appropriate responses for common questions: 0.8; YouChat and Copilot reliably appropriate responses for common questions: 0.6
Rahsepar et al. (2023)^45^	GPT-3.5; Bard; search engines	Medical knowledge	Lung	40; OE	3	Prevention; screening; and terminology commonly used GL for Lung Imaging Reporting and Data System (Lung-RADS) as reference. Presented to LLMs as well as Bing and Google search engines as control. Answers were reviewed for accuracy and consistency between runs.	2 Exp.	Accuracy on 4-point Likert-scale; Consistency (agreement between 3 runs)	GPT-3.5 responses were satisfactory with accuracy score 4: 0.708; Bard responses were suboptimal with accuracy score 4: 0.517; Bing responses were suboptimal with accuracy score 4: 0.617; Google responses were suboptimal with accuracy score 4: 0.55; GPT-3.5 and Google were most consistent; No tool answered all questions correctly and with full consistency
Musheyev et al. (2024)^30^	GPT-3.5; perplexity; ChatSonic;Copilot	Medical knowledge; summary/translation	Genitourinary	8; OE	NR	Top five search queries related to prostate; bladder; kidney; and testicular cancers according to Google Trends prompted to LLMs and Evaluated for quality; understandability; actionability; misinformation; and readability using validated published instruments.	NR	Multidimensional: Quality (DISCERN*); Understandability and Actionability (PEMAT-P*); Misinformation (5-point Likert-scale); Readability (FKGL)	LLMs responses had moderate to high information quality (median DISCERN score 4 out of 5; range 2–5) and lacked misinformation. Understandability was moderate (PEMAT-P understandability 66.7%; range 44.4–90.9%) and actionability was moderate to poor
Pan et al. (2023)^31^	GPT-3.5; perplexity; ChatSonic;Copilot	Medical knowledge; summary/translation	Skin; lung; breast; colorectal; prostate	20; OE	NR	Top five search queries according to Google Trends prompted LLMs and evaluated for quality; understandability; actionability; misinformation; and readability using validated published instruments.	2 Exp.	Multidimensional: Quality (DISCERN*); Understandability and Actionability (PEMAT-P*); Misinformation with GL as reference (5-point Likert-scale); Readability (FKGL*)	LLMs performed satisfactory with median DISCERN score: 5; median PEMAT-P understandability score: 0.667; median PEMAT-P actionability score: 0.2; and no misinformation. Responses are not readily actionable and are written at too complex a level for patients.
Huang et al. (2023)^32^	GPT-3.5; GPT-4	Medical knowledge	Radiation oncology	293 MCQ	NR	Radiology (ACR) radiation oncology exam Grey Zone cases are used to benchmark the performance of GPT-4.	1 Exp.	Multidimensional: Correctness; Comprehensiveness (4-point Likert-scale); Novel aspects not mentioned by experts’ hallucinations (“present” vs. “not present”).	GPT-4 outperformed GPT-3.5 with average accuracy: 0.788 vs. 0.621. Limitations deemed due to risk of hallucinations.
Nguyen et al. (2023)^33^	GPT-4; Bard	Medical knowledge	Various cancer entities	OE; SATA; PE	NR	Questions about cancer screening strategies were prompted in OE and SATA structure; authors compared differences in providing context or not.	2 Stud.	Accuracy for open-ended prompts (score range 0–2) and select-all-that-apply prompts (score range 0–1)	GPT-4 and Bard average score for open-ended prompts: 0.83 and 0.7. GPT-4 and Bard average score for select-all-that-apply prompts: 0.85 and 0.82. PE enhanced LLM outputs for OE prompts but did not improve SATA responses.
Iannantuono et al. (2024)^44^	GPT-3.5; GPT-4; Bard	Medical knowledge; summary/ translation	Immuno- oncology	60; OE	3	Evaluating questions to 4 domains of immuno-oncology (Mechanisms; Indications; Toxicities; and Prognosis)	2 Exp.	Accuracy (point-based scale 1–3); Relevance (point-based scale 1–3); Readability (point-based scale 1–3)	GPT-3.5 and GPT-4 number of answered questions: 1.0; Bard number of answered questions: 0.53. Google Bard demonstrated relatively poor performance. Risk of inaccuracy or incompleteness was evident in all 3 LLMs, highlighting the importance of expert-driven verification.
Liang et al. (2024)^34^	GPT-3.5; GPT-4; GPT-3.5 Turbo	Medical knowledge	Genitourinary	80; OE	3	Questions from urology experts were posed three times to both GPT-3.5 and GPT-4; Afterwards iterative fine-tuning with GPT-3.5 Turbo on the same question-set with and assessment of training outcomes.	NR	Binary accuracy (yes/no)	GPT-3.5 average accuracy: 67.08%: GPT-4 average accuracy: 77.50%; Both GPT-3.5 and GPT-4 were subject to instability in answering; GPT-3.5 Turbo stabilized average accuracy: 93.75%; With second iteration GPT-3.5 Turbo achieved 100% accuracy
Marchi et al. (2024)^35^	GPT-3.5	Medical knowledge	Oropharyngeal	68; OE	2	Questions on treatment; adjuvant treatment; and follow-up compared to GL (NCCN) as reference. Evaluated for sensitivity; specificity; and F1 score	2 Exp.	Binary accuracy (yes/no)	Overall sensitivity: 100%; Overall accuracy: 92%; Overall F1-score: 0.93; Overall precision: 91.7%.
Yeo et al. (2023)^36^	GPT-3.5	Medical knowledge; patient empowerment	Liver	164; OE	2	Questions regarding knowledge; management; and emotional support for cirrhosis and HCC and assessed for accuracy and emotional support capacity	2 Exp.	Accuracy (4-point Likert scale)	LLMs responses were satisfactory with an accuracy: 0.74; LLM had the best performance in basic knowledge; lifestyle; and treatment. LLM encourages patients to follow treatment strategies; offer emotional support; and recommend patients to seek sources such as support groups in a structured manner.
Hermann et al. (2023)^37^	GPT-3.5	Medical knowledge	Cervical cancer	64; OE	NR	Questions on prevention; diagnosis; treatment and QoL were drafted from p official patient forum websites and the authors’ clinical experiences and evaluated for correctness and comprehensiveness.	2 Exp.	Accuracy (4-point Likert scale)	LLM responses were satisfactory with correct and comprehensive: 0.531; correct and not comprehensive: 0.297; partially incorrect: 0.156; completely incorrect: 0.16; LLM performed best in “Prevention/QoL” and worst in “Diagnosis”
Lechien et al. (2024)^38^	GPT-4	Medical knowledge	Oropharyngeal	20; OE	2	Detailed patient history with head and neck cancer was evaluated for additional examinations; management; and therapeutic approaches and compared to the reference (TB decision).	2 Exp.	Multidimensional: AIPI Tool*	GPT-4 was accurate in 13 cases (65%). Mean number of treatment recommendations proposed by LLM: 5.15; Mean number of treatment recommendations proposed by tumour board: 4. Test–retest showed mostly consistent LLM outputs
Kuşcu et al. (2023)^39^	GPT-4	Medical knowledge	Oropharyngeal	154; OE	2	Questions from various sources: official patient forum; institutions; patient support groups; and social media. Topics: basic knowledge; diagnosis; treatment; recovery; operative risks; complications; follow-up; and cancer prevention.	2 Exp.	Accuracy (4-point Likert scale) Reproducibility (number of similar responses)	LLMs responses were satisfactory with an accuracy: 0.863. Reproducibility: 0.941 upon test–retest evaluation.
Chung et al. (2023)^47^	GPT-3.5	Translation/summary	Prostate	5; OE	3	Prompted to summarize five full MRI reports and evaluated for readability. Radiation oncologists were asked to evaluate the AI-summarized reports via an anonymous questionnaire.	12 Exp.	Accuracy (Likert-scale 1–5); Readability (FKGL*)	LLM was able to simplify full MRI reports at or below a sixth-grade reading level (9.6 vs. 5.0); Median word count was reduced from 464 (full MRI) to 182 (LLM). Summaries were deemed appropriate for patients.
Choi et al. (2024)^40^	GPT-3.5	Medical knowledge	Kidney cancer	10; OE	NR	FAQs drafted and evaluated for the service quality The survey was distributed to 103 urologists via email; and 24 urological oncologists.	103 Exp.	Service quality (SERVQUAL*)	Mean positive evaluation rate: 0.779; Positive scores for the overall understandability: 0.542; LLM could not replace explanations provided by experts: 0.708
Dennstädt et al. (2024)^41^	GPT-3.5	Medical knowledge	Radiation oncology	70 MCQ; 25 OE	NR	Multiple-choice questions about clinical; physics; and biology general knowledge and evaluated for correctness and usefulness	6 Exp.	Accuracy (5-point Likert scale); Usefulness (5-point Likert scale)	LLM valid responses in multiple-choice questions: 0.943; LLM very good responses in open-ended questions: 0.293
Wei et al. (2024)^42^	GPT-4	Medical knowledge; summary/translation	Oropharyngeal	49; OE	NR	Commonly asked questions about head and neck cancer were obtained and inputted into both GPT-4 and Google search engines.	2 Exp.	Quality (5-point Likert scale using EQIP Tool*) Readability (FRE*; FKGL*)	Google sources received significantly higher quality scores than LLM (4.2 vs. 3.6); No significant difference between the average reading ease score for Google and LLM (37.0 vs. 33.1) or the average grade level score for Google and LLM (14.2 vs. 14.3)
Lee et al. (2023)^43^	GPT-3.5	Medical knowledge; summary/translation	Oropharyngeal	NR	NR	Generated from presurgical educational information, including indications; risks; and recovery common surgical questions. Evaluated for thoroughness; Inaccuracy and readability and compared to search engine.	5 Exp.	Accuracy; Thoroughness (10-point Likert scale); Inaccuracy (number of errors); Readability (FRE*; FKGL*)	LLM and Google showed similar accuracy (mean 7.7 vs. 8.1) and thoroughness (mean 7.5 vs. 7.3), with few medical errors (mean 1.2 vs. 1.0). Readability was comparable between both tools. Experts preferred Google 52% of the time.

*OE* open-ended, *SATA* Select-All-That-Apply, *n.a.* not applicable, *c.i.* clinical information, *Exp* expert, *Rv* reviewer, *NR* not reported, *PE* prompt engineering, *GL* Guideline, *MCQ* multiple choice question, *NCI* National Cancer Institute, * validated assessment tools, *FRE* Flesch reading ease, FKRG Flesch–Kincaid reading grade, DISCERN, ServQUAL, PEMAT-P, EQIP and AIPI are validated tools.

### Application domains, cancer entities and medical tasks

The majority of studies explored the prompting of medical questions and analysing the output of the LLMs (*medical knowledge*, 32/34)^14–45^, while a smaller portion explored LLMs for patient compliance (*patient empowerment*, 1/34)^36^ or their use for translating or summarizing information for patients (*translation/summary*, 2/34)^46,47^. The majority of the included studies focused on gynaecological cancer (8/34)^17,20,21,26,27,31,33,37^, prostate cancer (8/34)^16–19,22,30,31,47^, oropharyngeal cancer (6/34)^28,35,38,39,42,43^, or lung cancer (5/34)^17,31,33,45,46^, with some publications covering various cancer entities (4/34)^14,23,32,41^. The included publications evaluated different tasks. The two most prevalent tasks were prompting a LLM to provide appropriate diagnostic (14/34)^16,18,19,22,28,29,31,32,34,36,37,39,45,46^ as well as treatment recommendations (30/34)^14–43^.

### Testing the encoded medical knowledge of LLMs

**Fig. 1:**
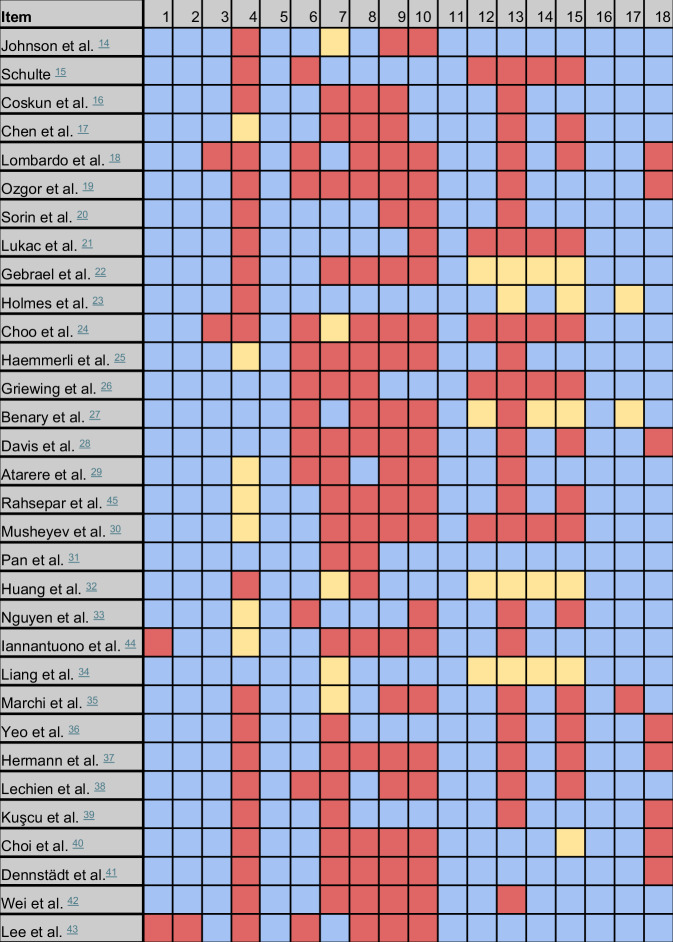
Reporting results of the eligible publications focusing on the application domain ‘Medical Knowledge’, two studies were excluded. Description of items is provided in the evaluation framework, please consider Table 1. Blue = reported, red = not reported, yellow = not applicable.

This section elucidates the methodology and results pertaining to the use of LLMs in the domain of medical knowledge. The developed evaluation framework encompasses the assessment of sources for generating inquiries, the language model utilized, the questioning procedure followed, and the methods for evaluating outputs. The results are illustrated in Fig. 1.

### Source of prompts

Various sources were used to generate input, i.e. questions for prompting. Sources encompassed official information forums (3/34)^14,16,18^, guidelines (7/34)^15,17,28,29,33,35,45^, frequently asked questions (FAQs) from various sources, social media, hospital websites and Google Trends^©^ (8/34)^19,30,31,36,37,39,40,42^, clinical cases (8/34)^20–22,24–27,38^, multiple-choice questions from medical exam banks (2/34)^23,32^ and some authors curated questions without reporting a specific source (4/34)^34,41,43,44^. English was predominantly used for prompting. A median of 51 questions (ranging from 8 to 293) were prompted. 61.8% of publications included the prompts and LLM outputs either as supplementary material or within the main text (21/34)^14,16–18,20–23,30–32,34–37,39–42,44,45^.

### Assessed large language model

All the studies have tested either GPT-3.5 or GPT-4. In total, 27 out of 34 studies evaluated the performance of GPT-3.5^14–18,20,21,23–32,34–37,40,41,43–47^, 11 out of 34 assessed GPT-4^19,22,23,32–34,38,39,42,44,46^, and 10 out of 34 examined multiple LLMs in comparison^23,27,29–31,33,34,44–46^. LLMs were utilized in their standard accessible versions via their website application or through an application programming interface (API).

Liang et al.^34^ reported the use of GPT-3.5 Turbo^48^, which is a payable service provided by OpenAI^©^ to fine-tune language models directed to specific tasks or domains. They employed a dataset comprising 80 questions related to renal clear cell carcinoma. The questions were designed with binary answers (true/false). To increase robustness, five distinct variations were added to each question, ensuring nuanced adjustments without deviating from the core essence of the original questions. Subsequently, the LLM's responses opposite to the ground truth were iteratively repeated until satisfactory outcomes were attained. By iteratively refining its performance on a foundational task set consisting of the binary designed questions, GPT-3.5 Turbo achieved an accuracy of 100% for the specific tasks^34^.

### Questioning procedure

A dedicated section within the evaluation framework thoroughly examines the reported questioning procedures (Table 1, *Items 5–11*). This aspect was the most under-reported in the included studies (Fig. 1). Only *Holmes et al*. provided a comprehensive account of their questioning procedure. Their detailed description included the number of questions, the design of questions (e.g. multiple-choice questions), test–retest cycles, and the use of prompt engineering prior to each cycle. Additionally, they provided source data along with prompt templates and initiated a new chat for each cycle to erase context^23^.

Test-retest reliability is a common psychometric parameter in the evaluation of scoring procedures^49^. This method was applied in 12 out of 34 of eligible studies^14,21,23,27,29,33–36,38,39,45^. Authors used test-retest cycles to evaluate the reliability and variability of LLM outputs to the posed questions, typically with a specific time interval in between tests.

Publicly accessible browser-based LLMs (e.g. GPT-3.5, Copilot, Gemini) offer a “new chat” function, which initiates a new conversation, erasing prior prompts and answers. Utilizing this function for each prompt was only employed in a few studies (9/34)^21,23,26,31–34,36,39^. The authors justified this method by noting that posing multiple continuous questions furnishes an LLM with context within a conversational framework. Continuous questioning can introduce context that may alter the performance of subsequent inquiries, as LLMs are proficient in-context learning. To mitigate these potential biases, it is advisable to initiate a “new chat” when assessing the “zero-shot” performance of LLMs concerning medical knowledge^50^.

The counterpart to the previously mentioned “zero-shot” prompting is conducting a conversational flow with a LLM. One out of 34 authors (*Schulte*^15^) reported conducting a continuous enquiry of the LLM, i.e. multiple questions within the framework of a conversation.

*Schulte*^15^ evaluated treatment recommendations for solid tumours and justified this approach to reduce variability and ensure the collection of data within a single session. The author allowed GPT-3.5 to tabulate possible therapies and used the National Comprehensive Cancer Network (NCCN) guidelines as a reference. GPT-3.5 was able to list 77% of the total possible therapies in concordance with the guidelines. The approach used by *Schulte* has been previously described by *Gupta et al*. as continuous or “fire-side” prompting, which can leverage more detailed LLM outputs because the conversation provides context to subsequent prompts^51^.

### Questioning procedure continued “Prompt Engineering”

Prompt Engineering (PE) was reported in 9 out of 34 studies^15,17,20,21,23,27,33,34,41^. Examples of PE were summarized in Table 2, showcasing a variety of methods described in the literature^52^. PE focuses on enhancing the outputs of LLMs by refining prompts and has the potential to improve the accuracy and consistency of their performance^53^. Nguyen et al. investigated the impact of providing context through PE on the performance of different prompts^33^. The authors found that applying context through PE increased the performance of Open-Ended (OE) prompts. Other researchers have explored various prompting style strategies, focusing on rephrasing prompts without altering their original intent (e.g. “What is the treatment for [X]?” vs. “How do you treat [X]?”). Chen et al. evaluated this approach using four different paraphrasing templates, which influenced both the number of treatments aligned with NCCN guidelines and the occurrence of incorrect suggestions^17^.

### Evaluation of LLM performance

**Fig. 2:**
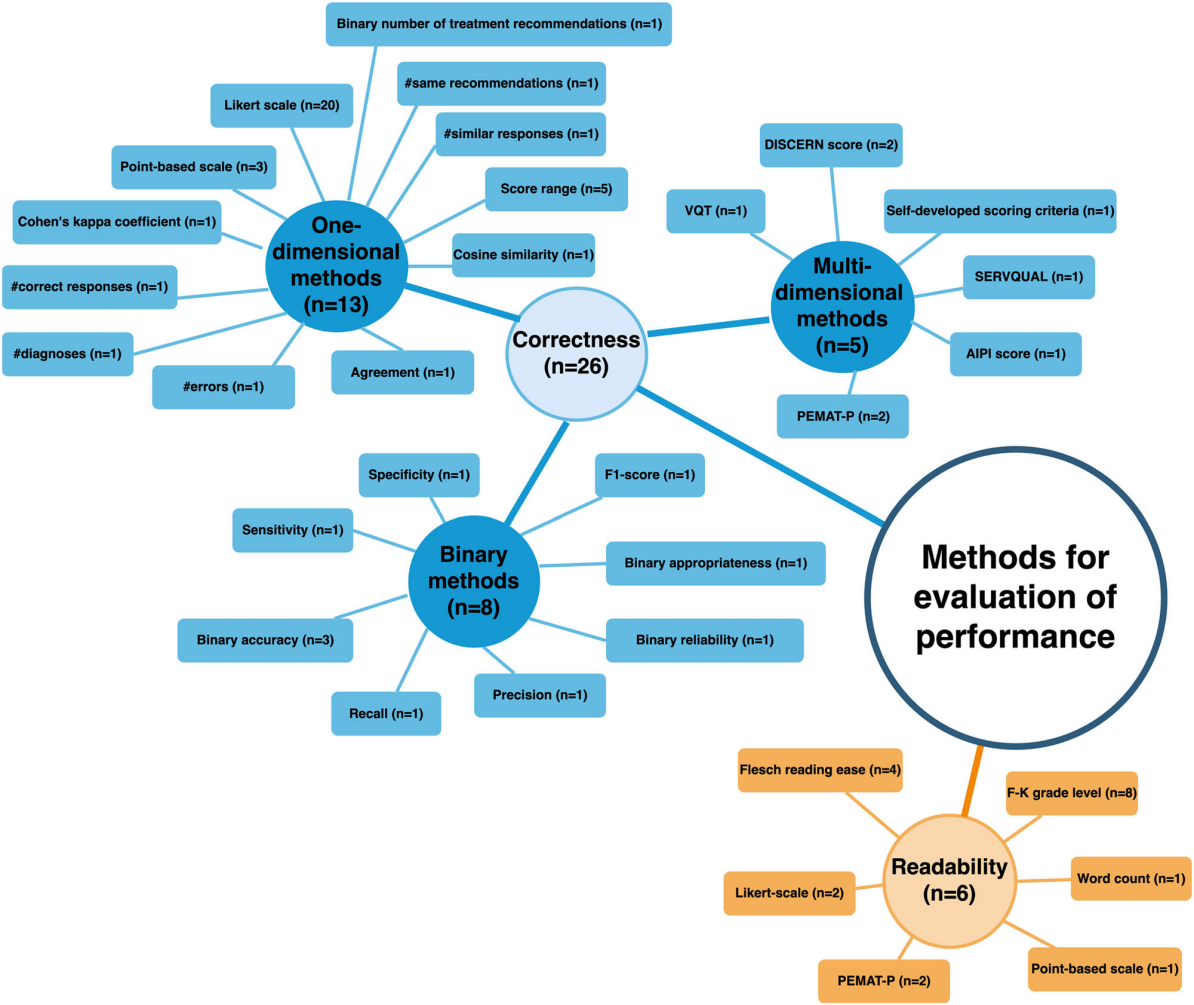
Categorization of the included grading methods based on the methods used for evaluation of the performance of the LLMs. The included metrics are grouped into two categories: those assessing correctness, and those assessing readability. The metrics dealing with correctness can be further divided into binary methods, one-dimensional methods, and multidimensional methods.

All publications included in the analysis reported an endpoint to describe the correctness or readability of LLM outputs as measures of performance. In total, 26 different terms used to assess correctness were identified (Fig. 2). The evaluation of LLMs based on the correctness of outputs was termed “grading”. Grading methods and the quantity of correct LLM outputs were among the most consistently reported subjects in the literature examined (Table 1, *Items 17–19*). The grading methods for correctness can be summarized into three groups: binary, one-dimensional, and multidimensional methods (Fig. 2).

The most popular grading methods for correctness were Likert-scales, with 20 studies employing this approach^16,18–21,24,25,27,28,30–32,36,37,39,41–43,45,47^. Among Likert-scales, the 4-point (*n* = 7)^18,24,32,36,37,39,45^, 5-point (*n* = 8)^19,20,28,30,31,41,42,47^ and 10-point (*n* = 3)^25,27,43^ scales were commonly used. The second most common was binary grading methods, where LLM outputs were graded as either correct or incorrect based on the source material (*n* = 8)^14,16,22,26,27,29,34,35^. Additionally, three publications evaluated multiple-choice questions^23,32,41^. Only a few studies (*n* = 5)^30,31,38,40,42^ utilized validated tools to assess LLM outputs. For example, Pan et al.^31^ and Musheyev et al.^30^ used the DISCERN tool to compare the performance of different LLMs. DISCERN is a validated tool developed for patients to assess the quality of written medical information on treatment choices from internet sources^54^. Lechien et al. created the artificial intelligence performance instrument (AIPI), a tool that provides a multidimensional scoring system to assess the performance of AI-generated outputs to medical questions^38^. It was specifically designed to assess the performance of generative artificial intelligence (GenAI) systems in clinical scenarios. The AIPI was tested for reliability and validity. The score comprises subscores assessing patient features, diagnosis, and additional examination. To mitigate subjectivity, the authors refrained from implementing Likert-scales. The findings demonstrated that the AIPI is a valid and reliable clinical instrument^38^. The AIPI was particularly validated on clinical scenarios involving the management of clinical ear, nose, and throat cases.

The performance of LLM outputs in terms of readability and understandability was assessed as a secondary endpoint by 8 out of 34 of the studies^14,28,30,31,42,43,46,47^. Flesch reading ease (FRE), Flesch–Kincaid grade level (FKGL), and the patient education materials assessment tool for printable materials (PEMAT-P) were used and are established instruments to assess the complexity of the text to estimate the understandability of a patient’s perspective^55,56^.

Only a few studies assessed the ability of LLMs to translate their outputs into understandable language for laypersons as a primary endpoint. Haver et al. demonstrated that the initial responses of GPT-3.5 to lung cancer questions were challenging to read^46^. To address this, simplified responses were generated using various LLMs, namely GPT-3.5, GPT-4, and Bard. LLMs succeeded in enhancing readability, with Bard showing the most improvement. However, the average readability of all simplified responses still exceeded an eighth-grade level, deemed too complex for the average adult patient^46^.

### Meta-analysis

**Fig. 3:**
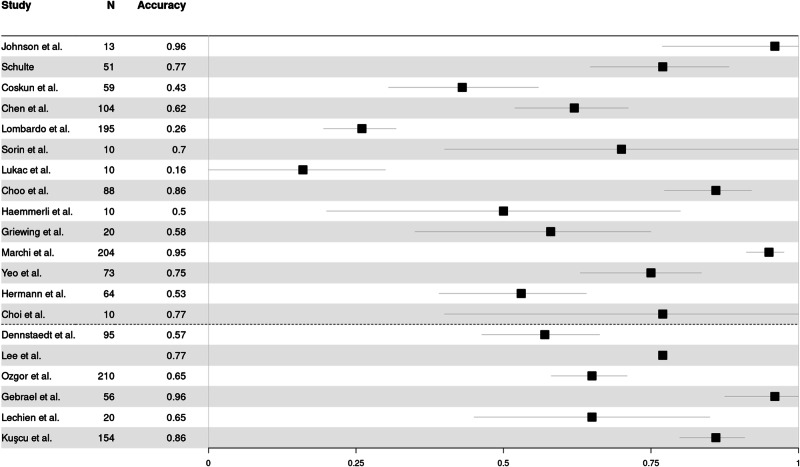
Forest plot showing the reported percentages of correct LLM outputs of studies assessing either GPT-3.5 or GPT-4. Above the dotted line used GPT-3.5, below the dotted line used GPT-4. *N* = number of questions evaluated with LLM.

**Fig. 4:**
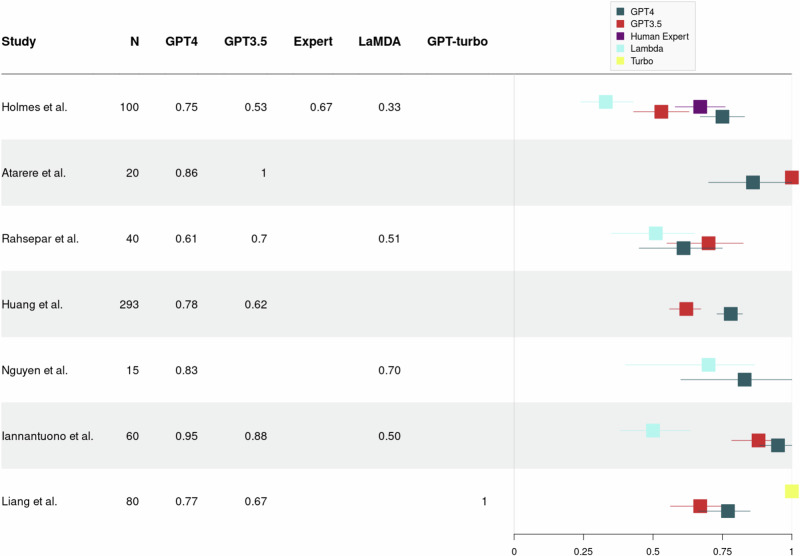
Forest plot showing the reported percentages of correct LLM outputs in publications that compared multiple language models in a benchmark. *N* = number of questions evaluated with LLM.

To illustrate the substantial variance that exists in the reported performance of LLMs in medQA, we conducted a formal and explorative meta-analysis. In total, 27 studies were eligible, evaluating the individual performance of one LLM or a comparative benchmark across multiple LLMs (as shown in Figs. 3 and 4). Studies evaluating single LLM assessed either GPT-3.5 or GPT-4, with mean accuracies across all studies of 63.6% (SD = 0.23) and 78.0% (SD = 0.16), respectively (see Fig. 3). The heterogeneity across studies yielded an *I*² value of 0%, indicating a substantial variability. In comparative assessments involving multiple LLMs, mean accuracy rates were 79% (SD = 0.10), 73% (SD = 0.17) and 51% (SD = 0.15) for GPT-4, GPT-3.5, and Bard (LaMDA), respectively, with a calculated *I*² value of 21% (see Fig. 4). The set of studies revealed an *I*² values of 0% and 21% underscoring the immense variability between studies which makes the results difficult to interpret.

## Discussion

This systematic review and meta-analysis aimed to analyse the current state of research, methodologies, and performance of LLMs in oncology. This review includes a diverse range of 34 eligible studies, most of which focused on testing the encoded medical knowledge of off-the-shelf LLMs. These studies primarily assessed LLM performances by evaluating their ability to answer oncological questions accurately and comprehensively. The formal meta-analysis revealed significant variability in reported LLM performances, emphasizing the influence that methodological inconsistencies, model capabilities, disease domains, in-context learning, fine-tuning and zero-shot prompting, as well as output rating strategies and metrics, have on assessment outcomes. Particularly notable was the absence of a standardized and validated evaluation system for LLM performance and significant heterogeneity in the questioning procedure, posing challenges to the comparability that must be addressed in forthcoming research.

The questioning procedure, as outlined by our evaluation framework, was among the most under-reported sections in the evaluated literature. This domain is termed prompt engineering (PE), which is paramount in the utilization of GenAI, particularly conversational agents like ChatGPT. Proper prompt design can enhance the performance of AI by guiding it to generate more accurate, relevant, and contextually appropriate responses. Wang et al. demonstrated how various prompt types distinctly affect LLM outcomes, and that reliability varies across consecutive prompts^53^. In turn, a potential source of inter-study heterogeneity is the difference in focus between evaluating “recall of medical facts” and “assessing medical reasoning capabilities”, as different studies may target one or both of these aspects, which lies within the structure of the prompts that are applied^53^. This underscores the need for precise prompting strategies to optimize outputs, as well as test-retest procedures to evaluate the reliability of LLMs. Future research should give a precise reporting of the prompting procedure and tactics employed, apply a test–retest procedure, as well as provide source data to increase transparency, facilitating that other researchers can recreate the experiment as proposed by our evaluation framework (see Table 1).

Hitherto, several AI-specific reporting guideline extensions have been added to aid standardized reporting in AI research (e.g. CONSORT-AI, TRIPOD-AI, STARD-AI or ESMO-GROW^57,58^). However, the current guideline extensions focus on machine learning and image-processing pipelines, making them unsuitable for LLM research. We addressed this gap with the development of our evaluation framework, which is based on the principles of QUADAS-2^11^. Due to a previous lack of LMM-specific reporting guidelines, we found no use of AI-specific reporting guidelines being utilized in the included studies. Most recently, the TRIPOD-LLM extension was published as a preprint, introducing a modular, task-specific approach addressing the unique challenges of LLM biomedical applications. This guideline should be considered for use in future publications^59^.

Another key factor contributing to the observed heterogeneity in LLM performances is the variation in model capabilities, as demonstrated by large-scale benchmarks such as MultiMedQA and ‘The Open Medical-LLM Leaderboard’^60,61^. These leaderboards documented significant variations in performance, not only due to inherent model differences but also across subspecialties. For example, the accuracy of Gemini Pro by Google ranged from 100% to 26%, for medical questions about gastroenterology and cardiology respectively^61^. In summary, inter-study variability is influenced by both the models themselves and the specific domains to which LLMs were applied to.

The high percentage (88.24%) of studies focusing on treatment recommendations highlights a strong interest in leveraging the encoded medical knowledge of LLMs, with potential applications such as patient education. Data from the 2018 ‘Health Information National Trends Survey’ showed that patients most frequently used the internet as their primary source for medical information^62^. Considering that, it is reasonable to expect that patients will use LLMs such as ChatGPT for the same purpose, which raises the question of how patients use LLMs to answer medical questions already. However, studies exploring patient interactions with GenAI are limited, and there is a notable gap between in-silico AI research, which shows promising results, and in-vivo research assessing AI integration into clinical workflows^63^. As a result, whether AI can effectively support cancer patients remains an open empirical question.

For clinical integration of AI, a fundamental concern is legal accountability in cases where erroneous decision recommendations are made^20,43,44^. It is important to establish a clear distinction between errors arising from faulty, incomplete user input or inaccuracies resulting from the interpretation of LLMs. A recent survey including 466 patients revealed that patients generally trust AI-assisted physicians for treatment and reported confidence in diagnoses made by AI only under physician control^64^. However, trust among physicians regarding LLM-generated treatment recommendations remains more divided. A study by Eppler et al. found that while 55.7% of respondents believe LLMs could play a role in supporting clinical decision-making, only 19.8% have incorporated ChatGPT into their practice. Trust in the accuracy of LLM-generated information is split, with 29.6% of participants expressing both trust and scepticism^65^. Handling accountability and safeguarding data privacy will emerge as challenges, particularly when navigating the complex landscape of diverse international regulatory frameworks^19,20,44^. It is crucial to implement robust mechanisms that ensure compliance with relevant data protection statutes across various jurisdictions. Data privacy-compliant LLMs, ideally deployed within secure hospital infrastructures, are essential to protect patient information while being effective tools in the clinical workflow. Enhanced systemic and patient-level oversight, supported by adequate resources, is essential for ensuring the quality and safety of LLM tools^66^. Gilbert et al. rigorously stated that LLMs and chatbots must receive approval as medical devices before implementation in clinical care^67^. Alongside regulatory approval, LLMs must be validated in real-world clinical settings. However, none of the reviewed studies explored LLMs in real-time clinical environments, highlighting a significant gap in the current research. Understanding how clinicians would interact with these models during live patient care is essential for their practical integration into clinical workflows. Future studies should prioritize real-time deployment to comprehensively evaluate the effectiveness and safety of LLMs in clinical practice.

Equally important is the process of optimizing and updating LLMs to be up-to-date with current research findings and clinical guidelines, especially in scenarios where LLMs would be legally classified as medical devices^25,44^. It is important to note that LLMs, like GPT-3.5 or GPT-4, are limited to chronological training cut-offs and are therefore not up-to-date. However, LLM can be enhanced using different methods. Liang et al. showed that the accuracy of GPT-3.5 could be increased to 100% when using an iterative fine-tuning process^34^. The authors demonstrated a method to maximize performance likely through overfitting, and ultimately focusing it on a specific task—answering questions exclusively about renal clear cell carcinoma (RCC). If the presented model we exposed to a different set of questions about RCC or questions outside the domain of RCC, the performance would likely decline, raising concerns about its clinical utility. A promising method of enhancing LLMs is called retrieval-augmented generation (RAG)^68^. RAG allows for the integration of a vector database that contains text-based knowledge (e.g. evidence-based guidelines), which communicates with a LMM-backbone to generate more accurate outputs. In a recent effort by our research group, we demonstrate that integrating urological guidelines into off-the-shelf LLMs using RAG results in superhuman performance on the European Urological Board exam, achieving an impressive 88% of correct answers. RAG not only increases accuracy but also allows for ready updates by replacing documents in the vector database. Most importantly, RAG adds a layer of explainability to LLMs, as the retrieved documents supporting an output can be displayed, making the responses more transparent and verifiable for the end-user^69^. Hence, RAG facilitates the incorporation of any latest evidence-based medical knowledge to improve the accuracy of LLMs and adds of a layer of explainability.

Current LLMs are powerful human language processors^70^, but their capabilities need to extend beyond text data to fully address the complexity of clinical oncology, which often includes visual data (e.g., diagrams, flow-charts). Future models must achieve optimal performance across all modalities—text and visual data—to become the comprehensive and effective tools in clinical oncology that are needed.

In conclusion, this systematic review and meta-analysis revealed that most current studies focus on the domain of medical knowledge and the evaluation of LLM performance in the capacity to answer oncologic questions correctly and comprehensively. The integration of LLMs in the medical sector presents a series of critical questions that demand to be addressed. Particularly notable was the absence of a standardized and validated evaluation system for language model performance and significant heterogeneity in the questioning procedure, posing challenges particularly to comparability and output evaluation that must be addressed. To enable comparability in the field, it is essential for authors to use standardized reporting guidelines for LMM-specific research. Addressing these challenges is essential for researchers to establish a safe, effective, and equitable integration of LLMs in clinical oncology.

This work must be reviewed considering its limitations, such as the source bias inherent to this review. All included publications were sourced exclusively from PubMed. While PubMed is a widely respected database for biomedical literature, relying solely on it may have limited the scope of this review. The use of the specific keyword “ChatGPT” may have introduced a selection bias. As a result, studies that examine similar technologies but do not use the term “ChatGPT” might have been inadvertently excluded. Despite these limitations, the extensive coverage of PubMed and the targeted approach provide a solid foundation to ensure a robust and comprehensive review. Another limitation is the potential for publication bias, which could result in information bias, as positive results are more likely to be published. This may lead to an overestimation of the effectiveness or impact of LLMs. High heterogeneity resulting in low observed *I*^2^ is considered as a limitation of our meta-analysis. However, we consider this acceptable as the analysis was intended to illustrate the variability stems from inconsistencies in current LLM research.

## Supplementary information

Supplementary Materials

## Data Availability

All data sources used in the study are publicly accessible, and detailed references are provided for each included study. Any additional data can be requested from the corresponding author.
